# Inflammatory response of microglia to prions is controlled by sialylation of PrP^Sc^

**DOI:** 10.1038/s41598-018-29720-z

**Published:** 2018-07-27

**Authors:** Saurabh Srivastava, Elizaveta Katorcha, Natallia Makarava, James P. Barrett, David J. Loane, Ilia V. Baskakov

**Affiliations:** 1 Center for Biomedical Engineering and Technology, University of Maryland School of Medicine, Baltimore, Maryland 21201 United States of America; 2 Department of Anatomy and Neurobiology, University of Maryland School of Medicine, Baltimore, Maryland 21201 United States of America; 3 Department of Anesthesiology and Shock, Trauma and Anesthesiology Research (STAR) Center, University of Maryland School of Medicine, Baltimore, Maryland 21201 United States of America; 4 Present Address: Department of Cellular and Molecular Medicine, University of California, San Diego, La Jolla, California 92093 United States of America

## Abstract

Neuroinflammation is recognized as one of the obligatory pathogenic features of neurodegenerative diseases including Alzheimer’s, Parkinson’s or prion diseases. In prion diseases, space and time correlations between deposition of disease-associated, pathogenic form of the prion protein or PrP^Sc^ and microglial-mediated neuroinflammation has been established. Yet, it remains unclear whether activation of microglia is triggered directly by a contact with PrP^Sc^, and what molecular features of PrP^Sc^ microglia sense and respond to that drive microglia to inflammatory states. The current study asked the questions whether PrP^Sc^ can directly trigger activation of microglia and whether the degree of microglia response depends on the nature of terminal carbohydrate groups on the surface of PrP^Sc^ particles. PrP^Sc^ was purified from brains of mice infected with mouse-adapted prion strain 22L or neuroblastoma N2a cells stably infected with 22L. BV2 microglial cells or primary microglia were cultured in the presence of purified 22L. We found that exposure of BV2 cells or primary microglia to purified PrP^Sc^ triggered proinflammatory responses characterized by an increase in the levels of TNFα, IL6, nitric oxide (NO) and expression of inducible Nitric Oxide Synthase (iNOS). Very similar patterns of inflammatory response were induced by PrP^Sc^ purified from mouse brains and neuroblastoma cells arguing that microglia response is independent of the source of PrP^Sc^. To test whether the microglial response is mediated by carbohydrate epitopes on PrP^Sc^ surface, the levels of sialylation of PrP^Sc^ N-linked glycans was altered by treatment of purified PrP^Sc^ with neuraminidase. Partial cleavage of sialic acid residues was found to boost the inflammatory response of microglia to PrP^Sc^. Moreover, transient degradation of Iκβα observed upon treatment with partially desialylated PrP^Sc^ suggests that canonical NFκB activation pathway is involved in inflammatory response. The current study is the first to demonstrate that PrP^Sc^ can directly trigger inflammatory response in microglia. In addition, this work provides direct evidence that the chemical nature of the carbohydrate groups on PrP^Sc^ surface is important for microglial activation.

## Introduction

Chronic neurodegeneration is an irreversible, fatal disorder of the central nervous system (CNS) that develops as a result of traumatic brain injury or age-related neurodegenerative maladies, including Alzheimer’s, Parkinson’s, ALS or prion diseases. Regardless of the specific disease or disease etiology, neuroinflammation, including activation of microglia and astrocytes, has been recognized as one of the most common pathogenic features of chronic neurodegeneration^1–4^. For elucidating mechanisms behind chronic neurodegeneration, prion disease offers several advantages over other neurodegenerative disorders. Prion diseases can be transmitted efficiently, not only to transgenic animals, but also wild type animals. Wild type mice infected with prions exhibit the full spectrum of neuropathological and biochemical features typically observed in naturally occurring prion diseases, including prion diseases of humans. Moreover, the time course of the disease in wild type animals is highly reproducible, coherent within animal groups and well-defined terminal stage.

While neuronal loss is a key pathological hallmark of prion diseases, activation and proliferation of microglia and astrocytes have also been recognized as obligatory features of the disease^5–7^. Studies that rely on unbiased whole genome expression documented that prion diseases are associated with a chronic neuroinflammation, with microglia being central to the disease process regardless of the prion strain or host^6^. The precise role of glia in chronic neurodegeneration has been under extensive debate and remains controversial^3,8–10^. Over the years, solid evidence has been put forward for both a protective phenotype and multiple inflammatory, neurotoxic phenotypes for microglia^7,11–20^. In the protective state, microglia are believed to be capable of neutralizing PrP^Sc^ while supporting neuronal function, whereas in inflammatory states, microglia attack and phagocytose neurons that are believed to be viable. Under pathological conditions, microglia have been shown to acquire a variety of activated phenotypes or functional states (in addition to M1 or M2 phenotypes), depending on the chemistry of the stimulus, prior activation, brain area and age of an organism^1,2,21,22^.

Previous studies that employed animals or post-mortem human brains revealed that activation and proliferation of microglia occur predominantly in the brain regions of PrP^Sc^ accumulation^6,11–13,23–27^. Moreover, widespread activation and proliferation of microglia and astrocytes was found to be at a much earlier stage than synaptic loss^5,7,27–29^, which is considered to be one of the earliest neuron-specific pathological sign that precedes neuronal loss^30,31^. By the clinical stage of the disease, microglial populations expand as much as 10-fold^3^. These results suggest that activation and chronic inflammation of microglia does not occur as a response to neuronal death. Nevertheless, it remains unclear whether neuroinflammation is secondary to neurodegeneration or a driving force of neurodegeneration. It is also unclear whether activation of microglia is triggered directly by a contact with PrP^Sc^ and what molecular features of PrP^Sc^ microglia sense and respond to that drive microglia to inflammation states.

PrP^Sc^ is a misfolded, highly aggregated, self-replicating state of the cellular sialoglycoprotein PrP^C^, which is modified posttranslationally with two N-linked glycans^32–36^. In intact PrP^Sc^ particles, the N-linked glycans are directed outwards exposing carbohydrate epitopes on PrP^Sc^ surface^37–39^. Our previous studies established that sialylation of PrP^Sc^ was important factor that determined the fate of prion infection^40–44^. In particular, we showed that PrP^Sc^ with reduced sialylation status did not cause prion disease in animals and that animals exposed to PrP^Sc^ with reduced sialylation via intracerebral or intraperitoneal routes remained free of prion infection for their life-time^40,42,43^. Moreover, sialylation of PrP^Sc^ was found to control trafficking of prions and their sequestration by secondary lymphoid organs upon peripheral exposure to prions^43^. In CNS, carbohydrate epitopes are recognized by several classes of carbohydrate-binding proteins, including C-type lectins, galectins, siglecs and selectins, which display selective preferences for specific carbohydrate groups^45^. Among diverse carbohydrate groups, sialic acid residues are of great importance, as they play essential role in recognition of pathogens and discriminating between self and non-self. Sialic acid residues occupy terminal positions on glycans and are found on all healthy mammalian cells (sialoglycocalyx), but are absent on most microbial pathogens^46,47^. Lack of terminal sialylation trigger response programs in cells of innate immune system, including microglia. A decline in sialic acid content on apoptotic or aging cells is recognized as part of apoptotic-cell-associated molecular patterns or ACAMPs and lead to activation of microglia and/or macrophages^9,48^.

Previous studies reported on space and time correlations between PrP^Sc^ deposition and microglia activation^6,11–13,23–27^. Studies conducted *in vitro* using microglial cell lines and scrapie brain homogenates assessed degradation of PrP^Sc^ by microglia cells^49,50^. However, it remained unclear whether PrP^Sc^ could directly trigger activation of microglia and whether the degree of microglia response to PrP^Sc^ depends on the nature of terminal carbohydrate groups on the surface of PrP^Sc^ particles. To address these questions, PrP^Sc^ was purified from brains of mice infected with mouse-adapted prion strain 22L or neuroblastoma N2a cells stably infected with 22L. We found that exposure of BV2 microglial cells and primary microglia to purified PrP^Sc^ triggered inflammatory responses characterized by an increase in the levels of TNFα, IL6, nitric oxide (NO) and expression of inducible Nitric Oxide Synthase (iNOS). Moreover, to test whether microglia response was mediated by carbohydrate epitopes on PrP^Sc^ surface, the level of sialylation of PrP^Sc^ N-linked glycans was altered by treatment of purified PrP^Sc^ with a neuraminidase. Partial cleavage of sialic acid residues was found to boost the inflammatory response of microglia to PrP^Sc^. These results provide direct evidence that the chemical nature of the carbohydrate groups on PrP^Sc^ surface is important for microglial activation.

## Results

### PrP^Sc^ deposition is accompanied by microglia activation in mice infected with 22L

**Figure 1:**
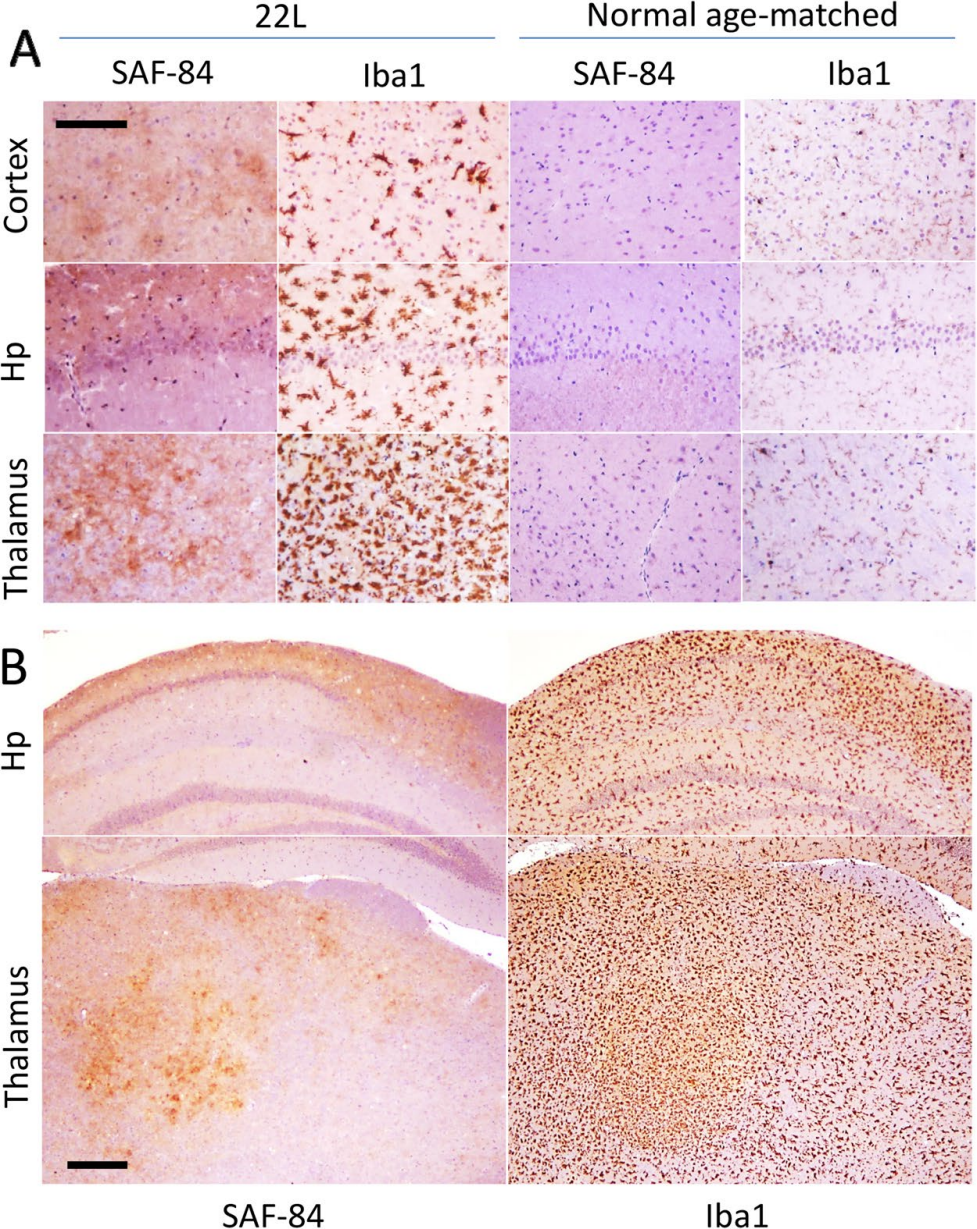
Immunohistochemistry of 22L-infected mouse brains. (**A**) Large magnification images of PrP^Sc^ deposition stained with SAF-84 antibody and activated microglia stained for Iba1 in cortex, hippocampus (Hp) and thalamus. SAF-84 and Iba1 staining of uninfected, age-matched controls is shown as references. Scale bar = 100 μm. (**B**) Low magnification images of PrP^Sc^ deposition stained with SAF-84 antibody and activated microglia stained for Iba1 in hippocampus (Hp) and thalamus. Scale bar = 300 μm.

To confirm that deposition of 22L PrP^Sc^ in brain is accompanied by activation of microglia, C57Bl/6 mice infected with 22L were euthanized at the terminal stage of the disease (145 days postinoculation) and their brains stained with anti-PrP antibody SAF-84 and an antibody to microglial marker Iba1 (Fig. 1). In 22L-infected mice, activated microglia were found in all anatomical regions with the strongest activation observed in thalamus, followed by hippocampus and cortex (Fig. 1). In contrast to the age-matched controls that showed microglia in a predominantly ramified state, in 22L-infected brains Iba1-positive cells displayed cell morphology typical for the inflamed states that are characterized by retracted processes and enlarged cell bodies (Fig. 1A). Moreover, consistent with previous studies^51^, a high density of Iba1-positive cells was observed in thalamus, a sign of active proliferation (Fig. 1A,B). While the areas with strong deposition of PrP^Sc^ showed the highest density of Iba1-positive microglia, activated microglia were widespread at the terminal stage of the diseases (Fig. 1B).

### Preparation of PrP^Sc^ with normal and altered sialylation status

22L PrP^Sc^ was purified from brains of terminally sick mice according to the procedure that was developed for obtaining pure and intact PrP^Sc^ with a high infectivity titer^52^ (Fig. S1A). Sialylation status was assessed using two-dimensional (2D) Western blots as described in our previous studies^41,53^. The sialylation pattern of purified PrP^Sc^ was found to be very similar if not identical to that of PrP^Sc^ in crude brain homogenate (Fig. S1B). For altering the sialylation status of PrP^Sc^, purified 22L PrP^Sc^ was treated with neuraminidase from *Arthrobacter ureafaciens* that has broad specificity and cleaves sialic acid residues regardless of their linkages^54^. The activity of neuraminidase was confirmed using treatment of bovine fetuin and denatured PrP^Sc^ as documented in our recent studies^54^. Analysis of sialylation status by 2D Western blot revealed a moderate shift of PrP charge isoform toward basic pH upon neuraminidase treatment demonstrating a partial cleavage of sialic acid residues (Fig. S1B). Incomplete desialylation is likely to be attributed to a highly aggregated nature of non-denatured brain-derived PrP^Sc^ and was consistent with our previous studies^40^. Purified, neuraminidase-treated 22L PrP^Sc^ will be referred to as ds-22L. As judged from Western blot, the amounts of purified PrP^Sc^ in 22L and ds-22L preparation stocks were equivalent to the amounts of PrP^Sc^ in 15% 22L scrapie brain homogenate (Fig. S1C).

For preparing mock material to be used as controls, healthy brains from age-matched mice were subjected to the same purification procedure as used for the purification of PrP^Sc^. Mock-purified materials will be referred to as NBH mock. To prepare control material for ds-22L PrP^Sc^, mock-purified NBH was treated with neuraminidase using the same procedure as used for 22L PrP^Sc^. Mock-purified NBH treated with neuraminidase will be referred to as ds-NBH mock.

### PrP^Sc^ induces proinflammatory responses in BV2 microglial cells

**Figure 2:**
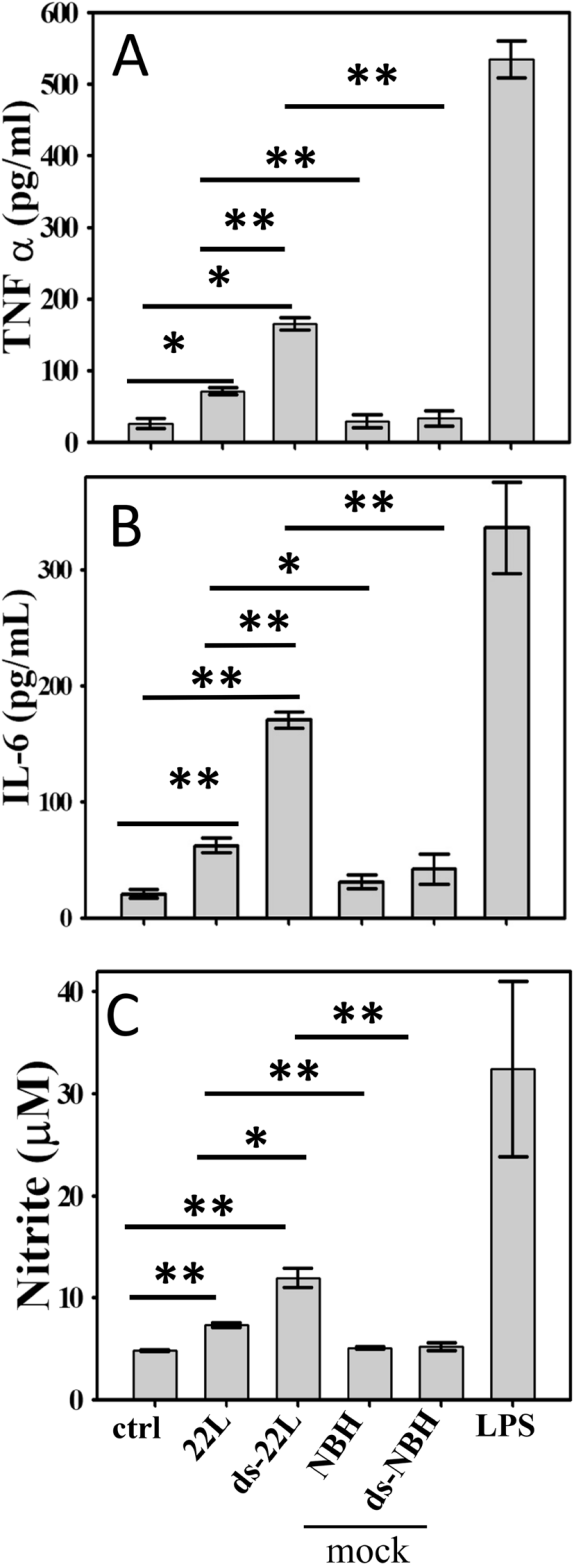
Analysis of PrP^Sc^-induced proinflammatory responses in BV2 cells. Levels of TNFα (**A**) IL6 (**B**) and NO (**C**) measured upon incubation of BV2 cells with purified 22L or ds-22L PrP^Sc^ for 18 hours. As negative controls, cells were treated with mock-purified NBH or ds-NBH, or cultured in the absence of treatment (ctrl). As a positive control, cells were treated with LPS (100 ng/ml). The final concentrations of 22L or ds-22L PrP^Sc^ in the cell culture media were equivalent to the amounts of PrP^Sc^ in 0.75% 22L scrapie brain homogenate. All experiments were performed three times, and the data are presented as the mean ± SD. Statistical significance (P) were calculated by Student’s t-test and indicated as * and ** for P < 0.05 and P < 0.005, respectively.

**Figure 3:**
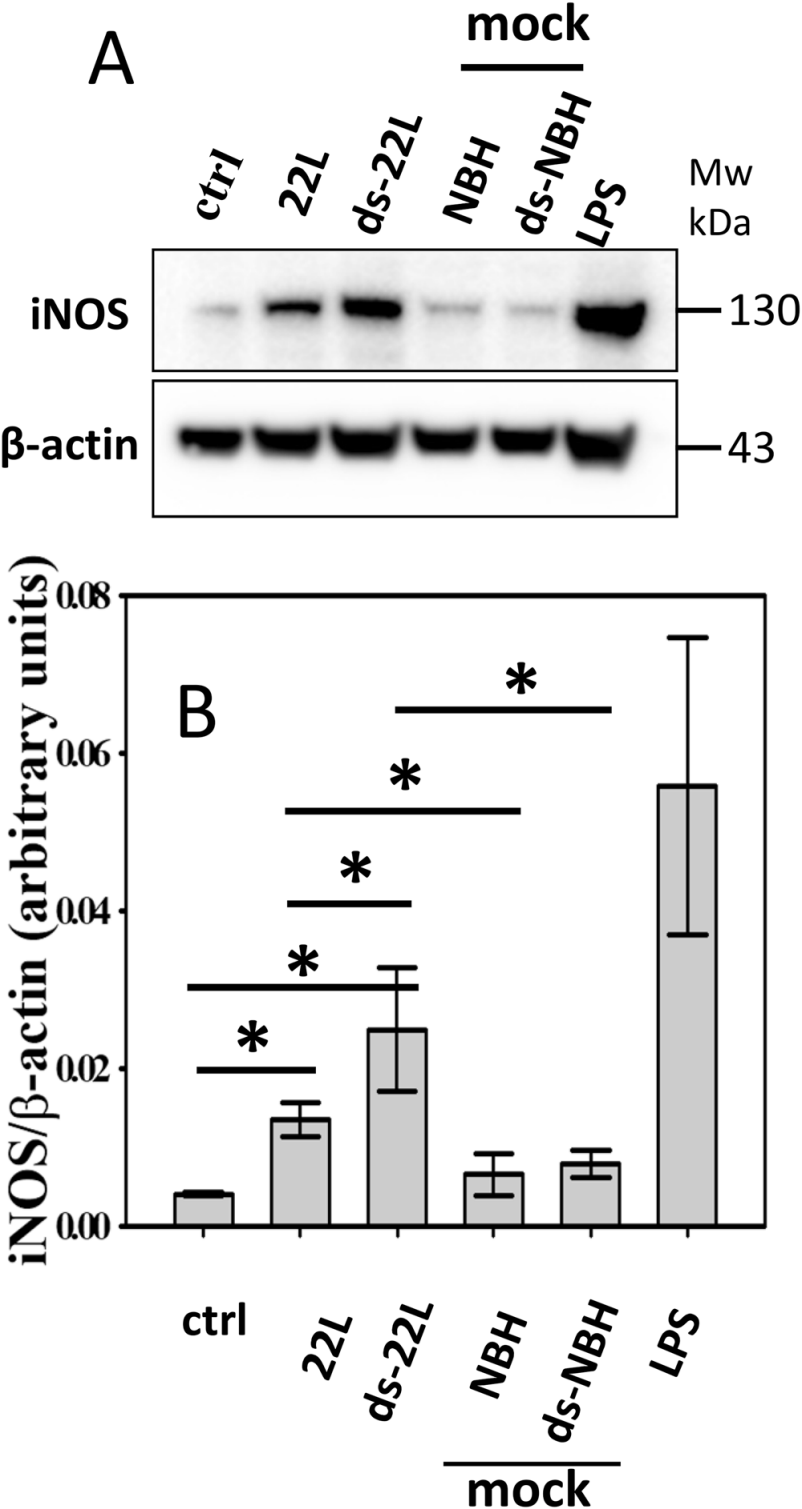
Analysis of iNOS expressions in BV2 microglia cells. (**A**) Western blot analysis of expression levels of iNOS in BV2 cells exposed to purified 22L or ds-22L PrP^Sc^ for 18 hours. As negative controls, cells were treated with mock-purified NBH or ds-NBH, or cultured in the absence of treatment (ctrl). As a positive control, cells were treated with LPS (100 ng/ml). The final concentrations of 22L or ds-22L PrP^Sc^ in the cell culture media were equivalent to the amounts of PrP^Sc^ in 0.75% 22L scrapie brain homogenate. The experiment was performed three times. Western blots probed with anti-β actin antibody served as protein loading control. Full-length Western blots are shown in Supplementary Fig. S4. (**B**) Densitometry analysis of iNOS expression in BV2 cells exposed to purified 22L or ds-22L PrP^Sc^. The data were normalized relative to the intensity of the β actin. The data are presented as the mean ± SD (n = 3 independent experiments). Statistical significance (P) were calculated by Student’s t-test and indicated as * for P < 0.05.

While widespread of inflamed, Iba1-positive microglia in prion-infected animals illustrates chronic neuroinflammation during prion infection (Fig. 1), however, it is not clear whether microglia is activated directly by PrP^Sc^. To test the hypothesis that PrP^Sc^ can directly trigger inflammatory responses in microglia and assess whether the amplitude of microglial responses is determined by the sialylation status of PrP^Sc^, BV2 microglial cells were cultured in the presence of purified 22L or ds-22L PrP^Sc^. The concentrations of 22L or ds-22L PrP^Sc^ in cell culture media were equivalent to PrP^Sc^ concentration in 0.75% 22L scrapie brain homogenate. As negative controls, cells were treated with mock-purified NBH or ds-NBH, or cultured in the absence of treatment. The gram-negative bacteria lipopolysaccharide (LPS) was used as a positive control for the inflammatory response. The inflammatory response was assessed by measuring production of TNFα, IL6 and nitric oxide (NO) (Fig. 2), or the expression levels of inducible Nitric Oxide Synthase (iNOS) (Fig. 3).

Upon treatment with purified 22L PrP^Sc^, BV2 cells displayed increased levels of TNFα, IL6 and NO (Fig. 2A–C), and expression levels of iNOS (Fig. 3A,B) in comparison to the untreated cells or mock-treated controls. Incubation with ds-22L PrP^Sc^ induced even stronger proinflammatory responses in BV2 cells relative to the cells exposed to 22L PrP^Sc^ with respect to the production of TNFα, IL6 and NO (Fig. 2A–C), and expression of iNOS (Fig. 3A,B). As expected, LPS induced much stronger proinflammatory responses, which is consistent with the acute responses triggered by LPS *in vivo* and confirms responsiveness of the cultured BV2 cells to the external proinflammatory stimuli.

To exclude the possibility that the response of BV2 cells to ds-22L PrP^Sc^ were due to presence of neuraminidase, the BV2 cells were treated with neuraminidases, heat-denatured neuraminidases and desialylation reaction buffer, and then tested for TNFα, IL6, NO and iNOS expression. In all tested conditions, the levels of TNFα, IL6, NO and iNOS were found to be the same as in untreated cells (Fig. S2). This experiment showed that BV2 cells did not exhibit any measurable inflammatory response when exposed to the amounts of neuraminidase used for preparing ds-22L PrP^Sc^ material.

The exacerbated inflammatory effects observed for the ds-22L relative to the 22L PrP^Sc^ was intriguing and was followed up by a dose response comparison of NO production in 22L- versus ds-22L-treated BV2 cells (Fig. S3). Within the range of PrP^Sc^ concentrations tested, which corresponded the amounts of PrP^Sc^ in 0.18–1.5% 22L scrapie brain homogenate, the stimulatory effects of ds-22L PrP^Sc^ were found to be higher relative to the corresponding effects of 22L PrP^Sc^ (Fig. S3). In fact, the levels of NO plateaued at high concentrations of 22L PrP^Sc^, whereas it continued to increase in ds-22L-treated cells. In summary, these results suggested that purified, brain-derived 22L PrP^Sc^ induced proinflammatory responses in BV2 microglia. The amplitude of the response increased upon a partial removal of sialic acid residues from the PrP^Sc^ N-linked glycans.

### Canonical NFκb activation pathway is involved in PrP^Sc^ induced inflammatory responses

**Figure 4:**
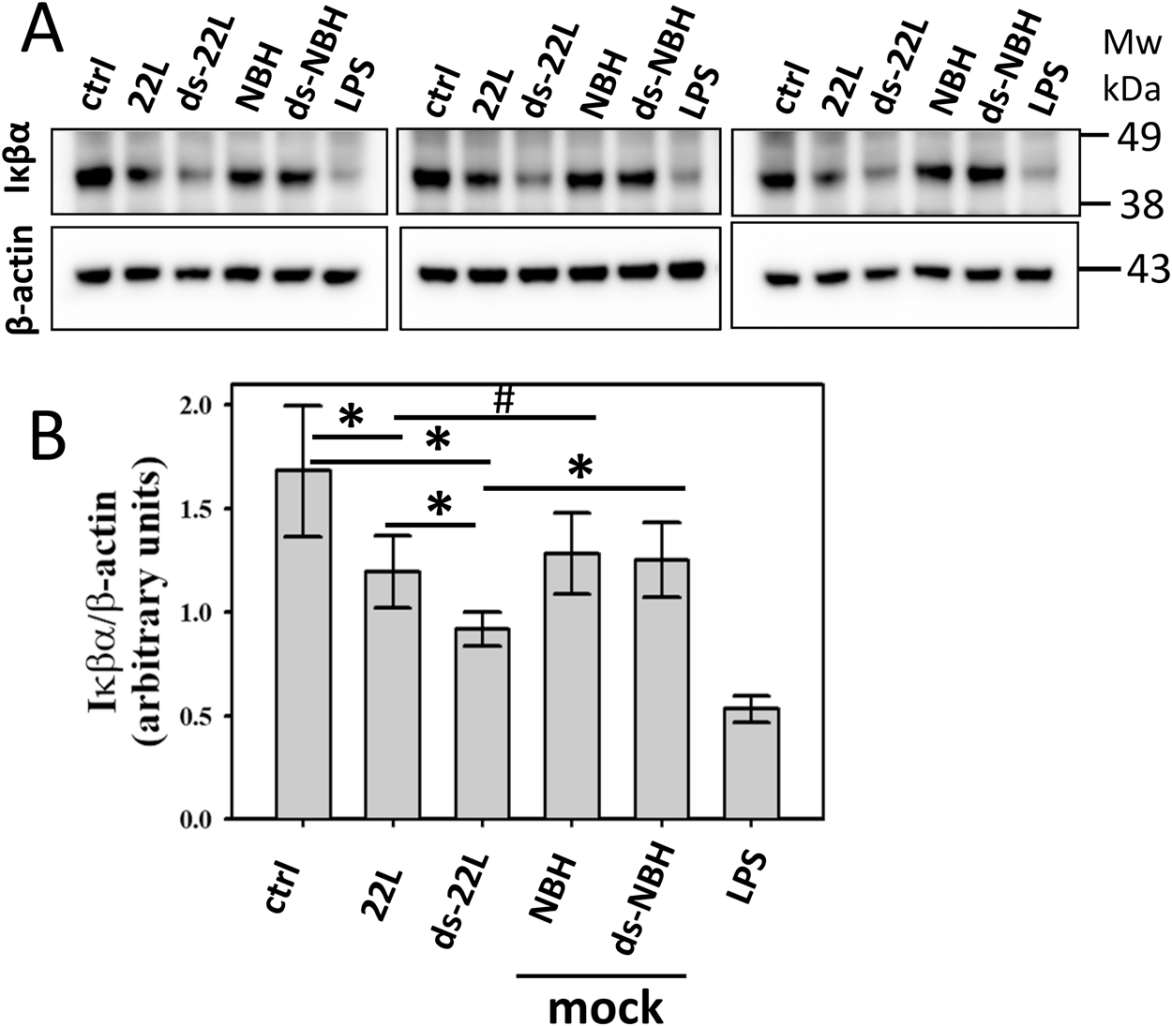
Analysis of Iκβα degradation in BV2 cells. Western blot analysis of cellular levels of Iκβα in BV2 cells exposed to purified 22L or ds-22L PrP^Sc^ for 20 minutes. As negative controls, cells were treated with mock-purified NBH or ds-NBH, or cultured in the absence of treatment (ctrl). As a positive control, cells were treated with LPS (100 ng/ml). The final concentrations of 22L or ds-22L PrP^Sc^ in the cell culture media were equivalent to the amounts of PrP^Sc^ in 0.75% 22L scrapie brain homogenate. The experiment was performed three times. Western blots probed with anti-β actin antibody served as protein loading control. Full-length Western blots are shown in Supplementary Fig. S5. (**B**) Densitometry analysis of cellular levels of Iκβα in BV2 cells exposed to purified 22L or ds-22L PrP^Sc^. The data were normalized relative to the intensity of the β actin. The data are presented as the mean ± SD (n = 3 independent experiments). Statistical significance (P) were calculated by Student’s t-test and indicated as * and ** for P < 0.05 and P < 0.005, respectively, and ^#^ for insignificant (P > 0.05) statistics.

iNOS over-expression and increased cytokine release in PrP^Sc^-treated BV2 microglial cells indicates a possible involvement of NFκB activation pathway in PrP^Sc^ induced inflammatory responses. To investigate whether a canonical or non-canonical NFκB activation pathway is involved, Iκβα degradation was examined in BV2 cells. A preliminary study revealed a drop in Iκβα levels in cells exposed to PrP^Sc^ with a maximum effect observed between 20 and 30 minutes after administration of PrP^Sc^. After exposure of cells for 20 minutes, a densitometry analysis of Western blots revealed statistically significant drop in Iκβα levels in ds-22L-treated cells relative to the mock-treated or non-treated controls (Fig. 4A,B). The levels of Iκβα in cells treated with 22L PrP^Sc^ were lower relative to the non-treated controls, but did not show statistically significant differences relative to the levels in corresponding mock-treated control cells (Fig. 4B). As expected, BV2 cells treated with LPS showed the strongest drop in Iκβα levels (Fig. 4A,B).

### The proinflammatory effect of PrP^Sc^ does not depend on the source of scrapie

To exclude the possibility that proinflammatory responses in microglia are attributed to brain-specific impurities that might be co-purified with brain-derived PrP^Sc^, next we decided to test PrP^Sc^ produced in cultured cells. Stable 22L scrapie infection was established in N2a cells (will be referred to as ScN2a cells), then PrP^Sc^ was purified from ScN2a cells and treated with neuraminidase from *Arthrobacter ureafaciens*. 2D Western blotting confirmed a significant shift in distribution of charge isoforms toward basic pH in neuraminidase-treated 22L purified from ScN2a cells relative to the mock-treated 22L of the same origin (Fig. S1B). In parallel to purification of PrP^Sc^ from ScN2a cells, non-infected N2a cells were subjected to the same purification procedure to generate mock-purified material (mock N2a) for negative controls. Mock-purified material treated with neuraminidase (referred to as mock ds-NBH) was produced as a negative control for ScN2a-deriveded ds-22L PrP^Sc^.

**Figure 5:**
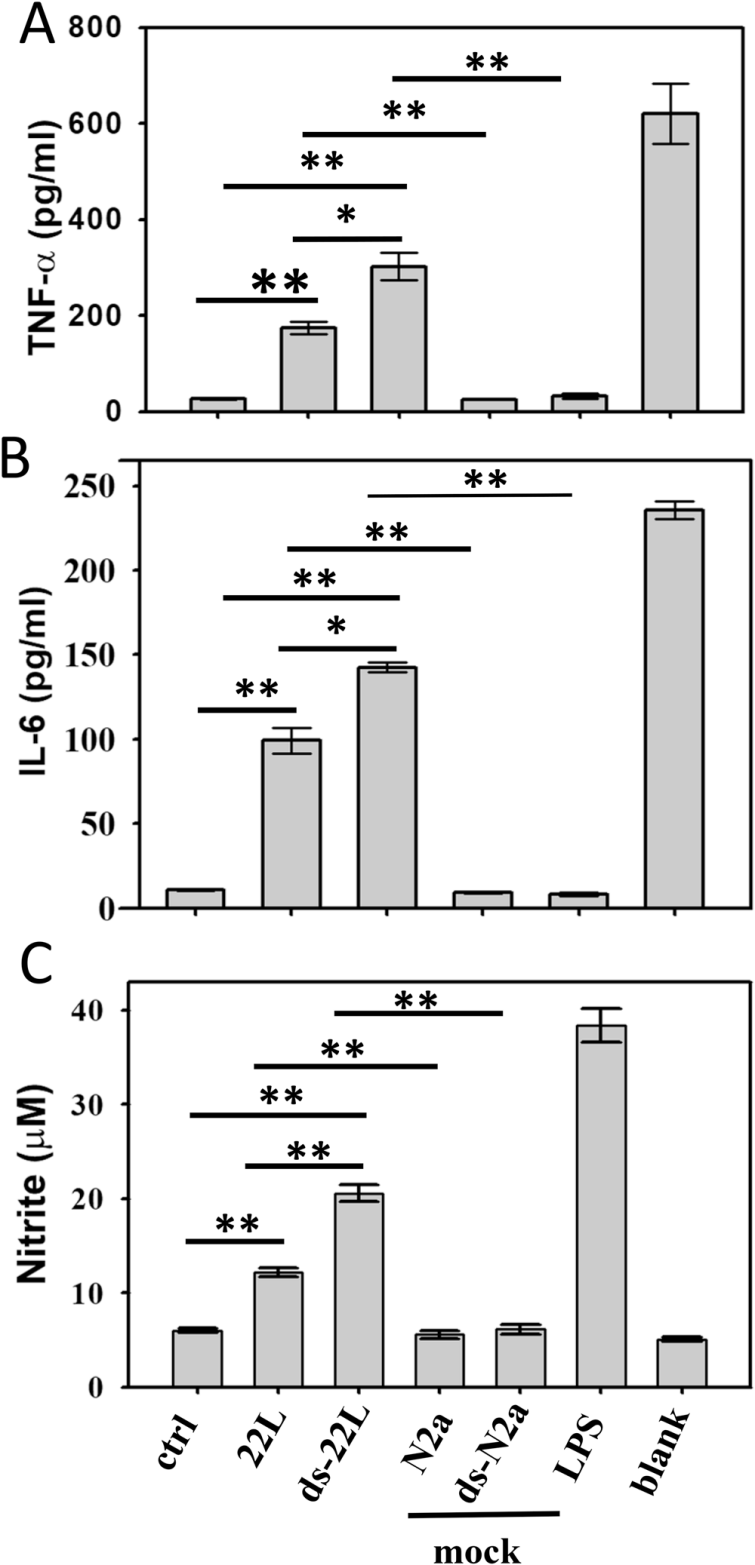
Analysis of proinflammatory responses in BV2 cells treated with cell-derived PrP^Sc^. Levels of TNFα (**A**) IL6 (**B**) and NO (**C**) measured upon incubation of BV2 cells with purified, ScN2a-derived 22L or ds-22L PrP^Sc^ for 18 hours. As negative controls, cells were treated with mock-purified N2a or ds-N2a, or cultured in the absence of treatment (ctrl). As a positive control, cells were treated with LPS (100 ng/ml). The final concentrations of ScN2a-derived 22L or ds-22L PrP^Sc^ in the cell culture media were equivalent to the amounts of PrP^Sc^ in 0.15% 22L scrapie brain homogenate. All experiments were performed three times, and the data are presented as the mean ± SD. Statistical significance (P) were calculated by Student’s t-test and indicated as * and ** for P < 0.05 and P < 0.005, respectively.

**Figure 6:**
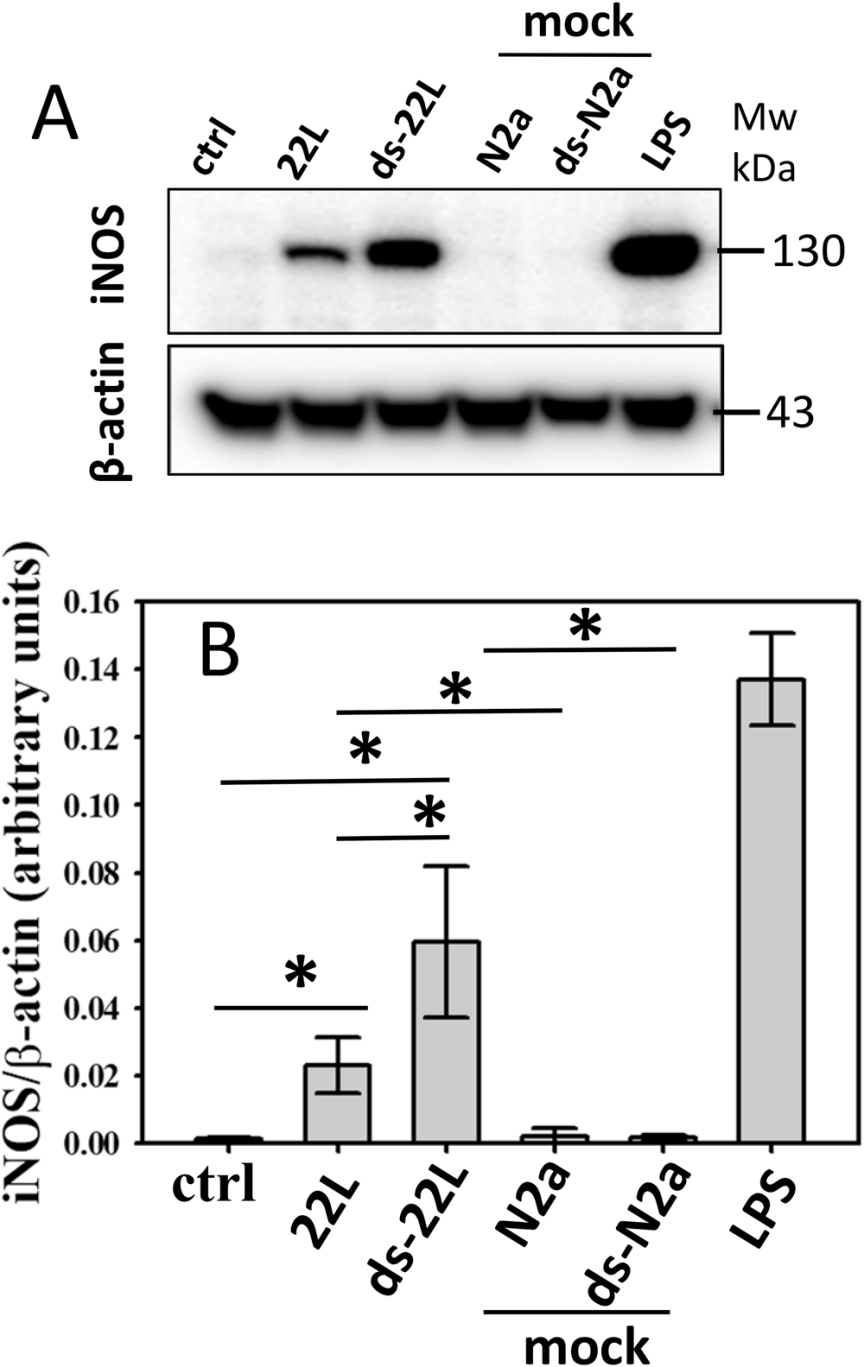
Analysis of iNOS expressions in BV2 cells treated with cell-derived PrP^Sc^. (**A**) Western blot analysis of expression levels of iNOS in BV2 cells exposed to purified, ScN2a-derived 22L or ds-22L PrP^Sc^ for 18 hours. As negative controls, cells were treated with mock-purified N2a or ds-N2a, or cultured in the absence of treatment (ctrl). As a positive control, cells were treated with LPS (100 ng/ml). The final concentrations of ScN2a derived 22L or ds-22L PrP^Sc^ in the cell culture media were equivalent to the amounts of PrP^Sc^ in 0.15% 22L scrapie brain homogenate. Western blots probed with anti-β actin antibody served as protein loading control. Three independent experiments were performed. Full-length Western blots are shown in Supplementary Fig. S4. (**B**) Densitometry analysis of iNOS expression in BV2 cells exposed to purified, ScN2a-derived 22L or ds-22L PrP^Sc^. The data were normalized relative to the intensity of the β actin. The data are presented as the mean ± SD (n = 3 independent experiments). Statistical significance (P) were calculated by Student’s t-test and indicated as * for P < 0.05.

The inflammatory response to ScN2a-derived PrP^Sc^ was examined by measuring the levels of TNFα, IL6, NO, and the expression levels of iNOS (Figs 5 and 6). The pattern of cell response to ScN2a-deriveded materials was very similar to the one observed for the brain-derived materials. Increased levels of TNFα, IL6, NO (Fig. 5A–C), and the expression level of iNOS (Fig. 6) were observed in BV2 microglial cells exposed to ScN2a-deriveded 22L PrP^Sc^ relative to the untreated cells or cells treated with mock-purified N2a. Again, incubation with ds-22L PrP^Sc^ induced even stronger proinflammatory responses in comparison to the cells treated with 22L PrP^Sc^ with respect to the production of TNFα, IL6, NO, and the expression of iNOS (Figs 5 and 6). These results demonstrated that PrP^Sc^ induced robust proinflammatory response regardless of PrP^Sc^ origin, and that removal of sialic acid residues enhanced its proinflammatory effect.

### Primary microglia cells exhibit similar pattern of proinflammatory responses to PrP^Sc^ as BV2 cells

**Figure 7:**
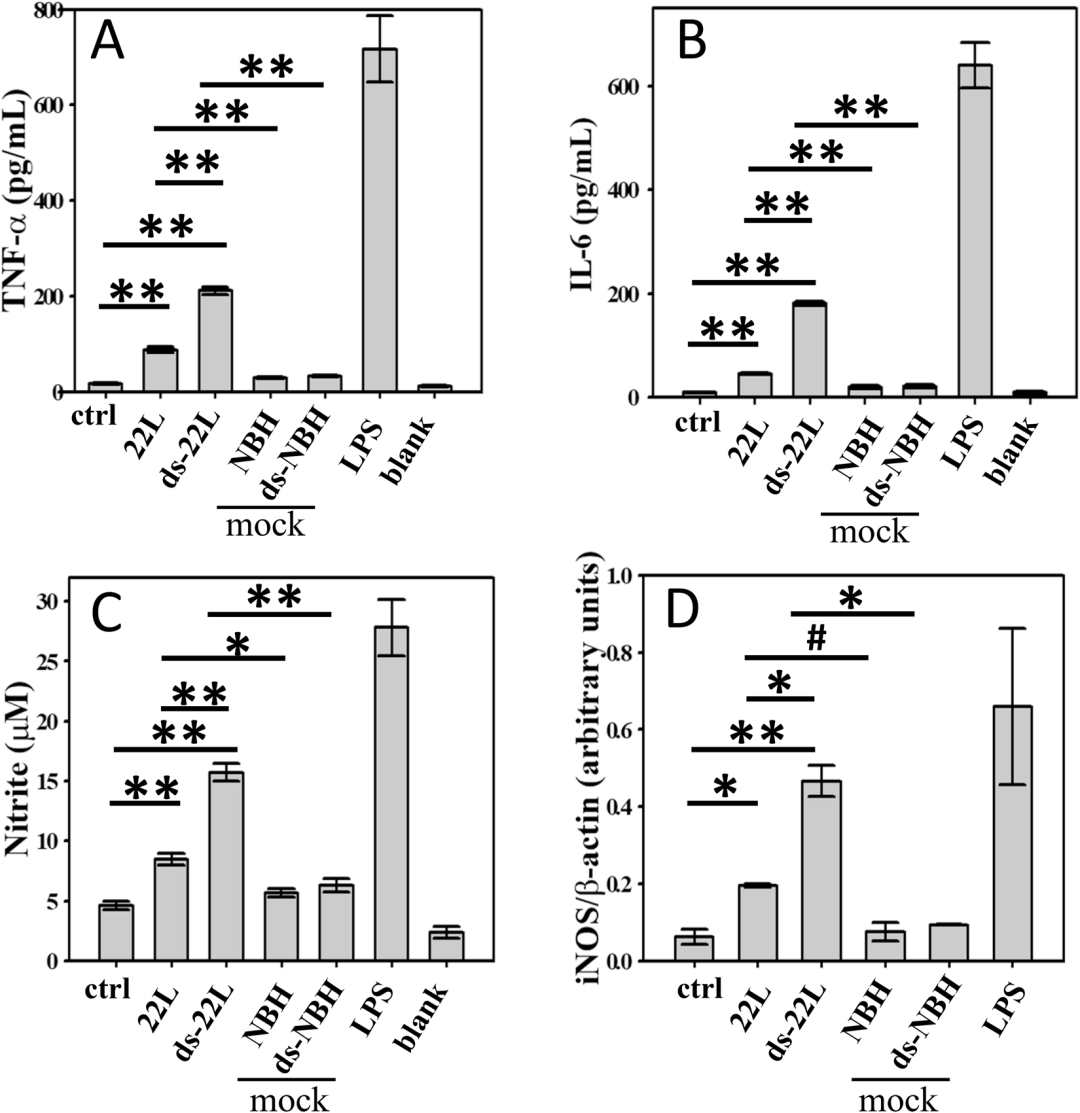
Analysis of PrP^Sc^-induced proinflammatory responses in primary microglia. Amounts of TNFα (**A**) IL6 (**B**) NO (**C**) and the expression levels of iNOS (**D**) measured upon incubation of primary microglia with purified, brain-derived 22L or ds-22L PrP^Sc^ for 18 hours. As negative controls, cells were treated with mock-purified NBH or ds-NBH, or cultured in the absence of treatment (ctrl). As a positive control, cells were treated with LPS (100 ng/ml). The final concentrations of 22L or ds-22L PrP^Sc^ in the cell culture media were equivalent to the amounts of PrP^Sc^ in 0.75% 22L scrapie brain homogenate. Western blots probed with anti-β actin antibody served as protein loading control for analysis of expression levels of iNOS in panel D. All experiments were performed three times, and the data are presented as the mean ± SD. Statistical significance (P) were calculated by Student’s t-test and indicated as * and ** for P < 0.05 and P < 0.005, respectively, and ^#^for insignificant (P > 0.05) statistics.

To test whether proinflammatory effects of PrP^Sc^ is BV2-specific or not, the immune response of primary microglia derived from neonatal C57Bl/6 mice were examined next. The levels of TNFα, IL6, NO, and the expression levels of iNOS were analyzed in cells treated with purified, brain-derived 22L or ds-22L PrP^Sc^ and compared to the mock-purified materials or nontreated control. The pattern of proinflammatory responses in primary microglia was found to be very similar to that of BV2 cells (Fig. 7A–D). Specifically, 22L PrP^Sc^ induced statistically significant increase in levels of all four inflammatory markers, when compared to non-treated cells or mock-treated controls (Fig. 7A–D). Upon administration of ds-22L PrP^Sc^, the levels of proinflammatory markers were found to be significantly higher than the levels of corresponding markers in cells treated with 22L PrP^Sc^ (Fig. 6). Again, LPS showed the strongest response (Fig. 6). These results established that PrP^Sc^ can directly trigger inflammatory response in primary microglia and the amplitude of microglia response depends on the sialylation status of PrP^Sc^.

## Discussion

Chronic neuroinflammation is recognized as one of the most common pathogenic features of prion diseases^5–7^. Yet, it remains unclear whether activation of microglia is triggered directly by PrP^Sc^. It is also not clear what molecular features of PrP^Sc^ microglia sense and respond to that drive microglia to activated proinflammatory states. To examine the effect of prions on activation of microglia, previous studies employed a short PrP-derived peptide 106–126 or recombinant PrP fragment 90–231 refolded *in vitro* into β-sheet reach conformation^55–59^. Since the peptide 106–126 and the recombinant fragment 90–231 do not recapitulate the structures of PrP^Sc^, nor do they possess PrP^Sc^-specific posttranslational modifications such as N-linked glycans, the extent to which the information generated with these fragments is relevant to prion diseases is not clear. The current study asked the questions whether PrP^Sc^ can directly trigger inflammatory responses of microglia and whether the degree of microglial activation depends on the nature of terminal carbohydrate groups on the surface of PrP^Sc^ particles.

The current studies revealed that PrP^Sc^ purified from brains of terminally sick mice infected with 22L prion strain induced proinflammatory responses in cultured BV2 microglial cells and primary microglia. The cell response was monitored by analysis of expression of cytokine TNFα, interleukin IL6, secretion of NO, and expression level of iNOS. Primary microglia showed very similar patterns of inflammatory responses to those observed for BV2 cells. Moreover, very similar patterns of inflammatory response were induced by PrP^Sc^ purified from brain and neuroblastoma cells arguing that microglia response is independent of the source of PrP^Sc^. Cross-comparison between brain- versus cell-derived PrP^Sc^ indicated stronger response of microglia toward cell-derived PrP^Sc^. This could be due to a less aggregated nature of the cell-derived versus brain-derived PrP^Sc^, i.e. larger exposed surface area of PrP^Sc^ purified from neuroblastoma cells than PrP^Sc^ from brain. In comparison to activation responses triggered by the toll-like receptor 4 ligand LPS, which induced very strong response in cultured cells and is known to trigger sharp acute response in animals^60^, purified PrP^Sc^ was mildly proinflammatory in nature. Such measured response correlates well with the sustained or chronic state of neuroinflammation in prion diseases and relatively slow progression of the prion disease in comparison to the acute response triggered by conventional pathogens. These results are consistent with the hypothesis that microglia cells are frequently exposed to PrP^Sc^ particles while surveying local CNS environment, and that exposure of microglia to mild but persistent inflammatory stimulus over the course of prion infection eventually results in widespread chronic inflammation.

Consistent with previous studies^6,11–13,23–27^, activated microglia were found to be widespread by the terminal stage of the diseases with the highest density observed in the areas of intense PrP^Sc^ deposition. The high density in the areas of PrP^Sc^ deposition suggests that PrP^Sc^ activate microglia and induce their proliferation. Alternatively, microglia might move toward the sites of PrP^Sc^ accumulation as they survey changes on CNS environment, the hypothesis that is difficult to test considering very dynamic nature of microglia. However, at the early stages of prion infection, activated microglia were not widespread and found predominantly at the early sites of PrP^Sc^ deposition^27^. The current work supports the hypothesis that PrP^Sc^ can directly induce activation of microglia.

Neuronal loss is considered as a key pathological hallmark of prion disease, yet activation and expansion of microglia is another essential feature of the disease^5–7^. The role of microglia in chronic neurodegeneration has been under extensive debates^3,8–10,16^. Previous studies established that suppressing microglia proliferation in prion diseases delayed the onset of behavioral symptoms and the terminal stage^7^. Moreover, a deficiency in Cx3cr1 receptor responsible for maintaining microglia in their resting state led to reduction in incubation time to disease^19^. These studies support the hypothesis that in prion diseases microglia acquire reactive, proinflammatory states, which contribute to disease progression. While the role of microglia in phagocytosing PrP^Sc^ has been documented^15,61^, microglia were also found to be more efficient in phagocytosing neuronal cells than PrP^Sc^ in animals infected with prions^62^. These studies suggest that microglia primed by PrP^Sc^ transition into aggressive, toxic phenotypes, which might drive chronic neurodegeneration. The current study is the first to demonstrate that PrP^Sc^ can directly trigger proinflammatory responses in microglia.

What molecular features of PrP^Sc^ microglia sense and respond to? Previously, we proposed that carbohydrate epitopes on PrP^Sc^ surface determine the range of potential PrP^Sc^-binding partners interacting with PrP^Sc^ and defining the nature of CNS response^44,63^. PrP^Sc^ particles are densely sialylated due to N-linked glycans, which expose sialic acid residues on PrP^Sc^ surface^53^. Sialylation of glycans and lipids plays an important role in biology. On a surface of mammalian cells, sialic acids act as a part of a “self-associated molecular pattern” helping the innate immune system to recognize “self” from “altered self” or “non-self”^9,46^. A decline in sialic acid content represents one of the molecular signatures of “apoptotic-cell-associated molecular patterns” found in apoptotic or aging cells, including neurons^9,45,48,64,65^. To ensure reactivity against apoptotic but not healthy neurons, the activation threshold of microglia must be carefully calibrated and controlled^66–70^. Bearing in mind that sensing of sialylation by microglia helps to discriminate between sialoglycocalyx of healthy cells versus aged or apoptotic cells, we were interested to test whether sialylation levels of PrP^Sc^ determine the degree of microglia activation. Indeed, consistent with this hypothesis, the current study revealed that drops in sialylation levels of purified PrP^Sc^ boost the proinflammatory responses observed in both BV2 cells and primary microglia. The current work provides direct evidence that microglia respond to changes in chemical nature of the carbohydrate groups on PrP^Sc^ surface.

To probe the mechanism behind PrP^Sc^-induced proinflammatory response, the current work examined degradation of Iκβα. Degradation of Iκβα activates NF-κB, a rapid acting transcription factor. NF-κB plays a key role in regulating the immune response to infection or cellular stress^71^, as it responds to a broad range of stimuli. We found a transient yet statistically significant drop in the levels of Iκβα upon treatments with ds-22L PrP^Sc^ or LPS. In 22L PrP^Sc^-treated cells, the levels of Iκβα dropped relative to the non-treated control within the time-frame examined, but not mock-treated controls. This result is consistent with other data in the current study suggesting that the inflammatory potential of partially desialylated PrP^Sc^ is higher than that of PrP^Sc^ with normal sialylation status. Activation of NF-κB upregulates expression of a number of genes including inducible Nitric Oxide Synthase or iNOS. iNOS is involved in immune defense mechanism by producing NO, a free radical with an unpaired electron. We observed an increase in both the expression levels of iNOS and the amounts of NO released upon treatment of microglia with PrP^Sc^. These results illustrate an induction in expression of iNOS rather than just enhancing its enzymatic activities. Elevated iNOS and NO were observed in response to PrP^Sc^ purified from brain or cultured cells. Moreover, both BV2 and primary microglia showed increase in iNOS and NO. The current results are consistent with a recent study that described an increase in NO and iNOS expression levels during the early stages of prion infection in animals^72^.

Chronic neuroinflammation is a common feature of many neurodegenerative diseases including Alzheimer’s disease. Yet, there is a considerable gap in our understanding of the relationship between age-dependent changes in glycosylation environment of CNS and etiology of neurodegenerative diseases. Several plausible mechanisms by which age-dependent changes in glycan metabolism, structure or composition might contribute to or even drive chronic neuroinflammation and related neurodegeneration. First, an age-dependent decline in total sialic acid content and sialoglycoproteins in CNS has been observed^73^. Whether these changes could prime microglia via altering its activation threshold, and set up a stage for chronic neuroinflammation remains to be determined. Second, key molecules involved in Alzheimer’s diseases including amyloid precursor protein or APP, BACE1, ADAM10, nicastrin, TREM2 and PrP^C^ are all sialoglycoproteins^74^. Modifications of the N-linked glycans of BACE1 were found to result in changes in its cellular localization and drastic loss of Aβ deposition^75^. Knocking out cellular neuraminidase 1 was found to have dramatic effect on proteolytic processing of PrP^C^ ^76^. However, it is unclear whether changes in glycan structure would alter cellular localization and processing pathways of APP and other glycoproteins involved in Alzheimer’s disease in a similar manner. Third, recent glycomics studies revealed changes in levels and structure of glycans of several glycoprotein groups including synaptic proteins, memory-associated proteins and serum glycoproteins in patients with Alzheimer’s disease^77,78^. However, it remains to be determined whether alterations in structures of glycans attached to the membrane or soluble proteins play a role in etiology of Alzheimer’s disease. Fourth, the expression of a sialic acid-binding lectin CD33, which is a risk factor of Alzheimer’s disease, was found to increase in microglia cells in patients with Alzheimer’s disease^79^. Increased expression of CD33 positively correlated with the increase in the amounts of Aβ plaques^79^. However, the question whether CD33 senses normal sialoglycocalix of healthy cells, disease-associated changes in carbohydrate environment, or both, still remains open. As carbohydrate patterns on PrP^Sc^ surface can be manipulated, prions offer a valuable model system for examining the relationship between changes in carbohydrate environment and glial responses.

## Conclusion

The current study established that exposure of BV2 microglial cells and primary microglia to purified PrP^Sc^ triggered inflammatory responses characterized by an increase in the levels of TNFα, IL6, NO and expression of iNOS. Moreover, partial cleavage of sialic acid residues was found to boost the inflammatory response of microglia to PrP^Sc^. These results provide direct evidence that PrP^Sc^ can directly induce proinflammatory response in microglial cells and that the chemical nature of the carbohydrate groups on PrP^Sc^ surface is important for microglial activation.

## Methods

### Ethics statement

This study was carried out in strict accordance with the recommendations in the Guide for the Care and Use of Laboratory Animals of the National Institutes of Health. The animal protocol was approved by the Institutional Animal Care and Use Committee of the University of Maryland, Baltimore (Assurance Number A32000-01; Permit Number: 0215002).

### Animals and immunohistochemistry analysis

C57BL/6 mice were inoculated with 10% 22L scrapie brain homogenate prepared in PBS. The inoculum was dispersed by 30 sec indirect sonication at ~200 watts in the microplate horn of a sonicator (Qsonica, Newtown, CT) immediately before inoculation. Each C57BL/6 mouse received 20 μl of inoculum intracerebrally, under 2% isoflurane anesthesia. After inoculation, animals were observed daily for disease using a ‘blind’ scoring protocol and euthanized at the terminal stage of the disease (145 days postinoculation, 197 days old). Normal, age-matched control mice were 212 days old. Formalin-fixed brain halves divided at the midline (left hemisphere) were processed for immunohistochemistry staining. Blocks were treated in formic acid (96%) before being embedded in paraffin. For detection of disease-associated PrP^Sc^, blocks were pretreated by 15 min hydrated autoclaving at 121 °C in trisodium citrate buffer, pH 6.0 with 0.05% Tween 20, followed by 5 min in 88% formic acid. Anti-prion antibody SAF-84 (Abcam, Cambridge, MA) or rabbit anti-Iba1 (Wako, Richmond, VA) were used for staining, as indicated.

### Purification of PrP^Sc^ and treatment with neuraminidase

PrP^Sc^ materials were purified from brains of terminally ill C57BL/6 mice or scrapie infected N2a cells using previously published procedure with a minor modification^52^. In the final resuspension step, the 0.1% w/v sarcosyl solution was substituted with water to exclude detergent interference with subsequent enzymatic treatments and cellular assays. N2a cells stably infected with 22L were collected in 1xPBS, lysed by 30-s sonication (Misonix S-4000 microplate horn, Qsonica), and subjected to the same purification protocol as brain-derived material. To prepare partially desialylated 22L (ds-22L), purified brain- or cell-derived 22L PrP^Sc^ was treated with 800 units/ml of neuraminidase *Arthrobacter ureafaciens* (New England Biolabs, cat #P0722L) in the presence of GlycoBuffer1 supplied by enzyme manufacturer at 37 °C with shaking for at least 12 h as previously described^54^. The amounts of PrP^Sc^ in preparations of purified 22L and ds-22L were estimated by Western blot and subsequently adjusted to prepare stocks of 22L and ds-22L of equal concentrations. To prepare mock-purified materials, the same procedures used for purification of PrP^Sc^ were used for purifying mock materials from brains of uninfected age-matched C57BL/6 mice or uninfected N2a cells.

### BV2 and primary microglia cell cultures

Murine microglial BV2 cell line was maintained in Dulbecco’s modified Eagle’s media (DMEM, Corning Cellgro, cat #10-013CV) supplemented with 10% horse serum (cat #26050-088, Life Technologies) and 1% antibiotics (cat #15240-062, Life technologies) at 37 °C with 5% CO_2_ in humidified incubator (VWR, model# VWR51014995) as previously described^80^.

Primary mouse microglia were prepared from postnatal day one C57BL/6 mouse pups (P0) as previously described^81^. Briefly, pups were decapitated and whole brains were harvested and homogenized in cDMEM media containing 10% FBS (cat #10082-147, Life technologies) and 1% antibiotics (cat #15240-062, Life technologies). Mixed glial cells were incubated overnight, then cocktail of growth factors was added to the final concentrations 10 ng/ml M-CSF and 10 ng/ml GM-CSF (cat #416-ML-010 and 415-ML-010 respectively, R&D system). Cells were kept without changing media for 8 to 10 days to facilitate microglial proliferation. Mixed glial cell cultures were shaken at 120 rpm at 37 °C for 90 min, then detached microglia cells were separated and seeded at 100 × 10^3^ cells per well in 24-well plates in cDMEM media for further experiments.

### Stimulation of BV2 cells and primary mouse microglia with purified PrP^Sc^

BV2 cells were seeded at 50 × 10^3^ cells per well in 24-well plates (Corning costar, Cat #3524) and incubated overnight to achieve 70–80% confluency. Media was replaced with a fresh 200 µl DMEM media supplemented with 10% Horse serum and 1% antibiotics (cat #15240-062, Life technologies). Unless specified, cells were incubated in triplicates with 10 µl of purified PrP^Sc^ or mock-purified materials for 18 hr at 37 °C and 5% CO_2_ in DMEM media supplemented with 10% HS and 1X antibiotics. Primary microglia cells seeded in 24-well plates were incubated with 10 µL of purified PrP^Sc^ or mock-purified materials for 18 h in 200 µL cDMEM media (10% FBS, 1% antibiotic (cat #15240-062, Life technologies) at 37 °C and 5% CO_2_ in a humidified chamber. Under standard conditions, the final concentrations of brain- and cell-derived PrP^Sc^ in the cell culture medium were estimated to be equivalent of PrP^Sc^ in 0.75% 22L scrapie brain homogenate or 0.15% 22L ScN2a cell lysate. Cell cultures treated with 100 ng/ml lipopolysaccharides (from *E*. *coli* 0111:B4, Sigma, cat #L3012) served as positive controls of inflammatory response. After incubation with PrP^Sc^ for 18 hours, supernatants and cells were collected and used for analysis of nitrite oxide, TNFα, IL6 and iNOS.

### Analysis of nitrite oxide

Nitric oxide production was analyzed using Griess Reagent (cat # G4410-10G, Sigma) using procedure published elsewhere with slight modifications^82^. Briefly, cell media collected from BV2 cells or primary microglia was centrifuged at 500 g for 2 mins to remove cell debris if any, then 70 µl of supernatant was added to 70 µl Griess reagent in a clean 96-well plates (Cell Star, Greiner Bio-One, cat #655180), incubated for 5 min in darkness at room temperature, and absorbance was recorded at 548 nm and 595 nm using iMark microplate reader (cat #1681135, Bio-Rad). All samples were analyzed in triplicates. As control for absorbance by media components, 70 µl of fresh DMEM media (10% HS, 1X antibiotics) was mixed and incubated with 70 µl Griess reagent in parallel. The amounts of nitrite oxide was quantified using a calibration curve build using freshly prepared sodium nitrite (Sigma, cat #237213) and Griess reagent.

### Analysis of cytokine secretion

To analyze production of cytokines, cell cultures were treated with purified PrP^Sc^ or mock-purified materials for 18 h. The supernatants were diluted 4-fold using sterile PBS, pH 7.4 (Gibco, cat #10010-023) and supplemented with protease inhibitor cocktail (Roche, cat #04693159001). Productions of IL6 and TNF-α were quantified by ELISA using mouse IL6 and TNF-α kits according to the manufacturer’s protocols (R&D systems, cat #M6000B and cat #MTA00B, respectively). All samples for ELISA were prepared in triplicates; the experiments were repeated three times.

### Analysis of iNOS expression

To analyze iNOS expression, cells in each well were lysed in 200 μL lysing buffer MPER (Thermo Scientific, cat #78501) supplemented with protease inhibitor cocktail (Roche, cat #04693159001), then homogenized by brief sonication for 30 seconds (Misonix S-4000 microplate horn, Qsonica), supplemented with 4X SDS buffer (200 mM Tris-HCL, pH 6.8, 8% SDS, 40% Glycerol, 4% beta mercaptoethanol, 50 mM EDTA, 0.08% bromophenol blue) and incubated in boiling water for 10 minutes. Samples were analyzed using precast SDS-PAGE NOVEX gels (Thermo Fisher, cat #NP0343BOX), immunoblotted as described elsewhere and stained using anti-iNOS antibody (BD Biosciences, cat #610328) at 1:3000 dilution overnight. Western blots were developed using chemiluminiscence substrate (Thermo Scientific, cat # 1859674 & 1859675) and visualized using a FluorChem M system (ProteinSimple). The signal intensities were digitized using AlphaView software (ProteinSimple). Blots probed with anti-β actin antibody (Sigma cat #A5441) served as protein loading controls. Data from three independent experiments were used for densitometry analysis of changes in iNOS expression.

### Neuraminidase treatment of BV2 cells

BV2 cells were cultured in 24-well plates to 70–80% confluency and then treated either with 20 µL of sample containing of either (i) 800 units/ml neuraminidase from *Arthrobacter ureafaciens* (cat #P0722L, New England Biolabs), (ii) 800 units/ml heat-denatured neuraminidase, or (iii) 1x GlycoBuffer1 neuraminidase buffer supplied by manufacturer with the enzyme (cat #P0722L, New England Biolabs). Supernatants and cells were collected and analyzed for accumulation of NO, IL6, TNF-α or expression levels of iNOS as described above. Untreated and LPS-treated cells served as negative and positive controls, respectively. Experiments were done in triplicates, and β actin served as loading control for iNOS.

### Analysis of Iκβα degradation

In preliminary experiment, a time dependent degradation of Iκβα was established. Briefly, BV2 cells were cultured in 24-well plates to 70–80% confluency, then treated with brain-derived 22L PrP^Sc^, ds22L PrP^Sc^ or mock-purified materials for 10, 20, 30, 45 or 60 minutes. Cells treated with DPBS (Cat #INV-14190144, Life Technologies) or LPS (100 ng/ml, *E*. *coli* 0111:B4) served as negative and positive controls, respectively. Cells were harvested in 200 µl using the cell lysis buffer MPER (Cat #78501, Thermo Scientific), then supplemented with 4X SDS buffer, incubated in boiling water for 10 minutes and analyzed using precast SDS-PAGE NOVEX gels (Thermo Fisher, cat #NP0343BOX). Blots were stained using anti-Iκβα antibody (cat #9242S, Cell Signaling Technology) at 1:1000 dilution. Blots were re-probed with anti-β actin antibody, which served as protein loading control, using reblotting buffer (Millipore cat #2502). For analysis of Iκβα degradation at the fixed time point, BV2 cells cultured in 24-well plates were treated with brain-derived 22L PrP^Sc^, ds-22L PrP^Sc^, mock-purified materials, DPBS or LPS for 20 minutes. And analyzed as described above. β actin served as loading controls.

### 2D Electrophoresis

Samples of 25 µL volume heated for 10 min in a boiling water bath in the presence of gel loading buffer were solubilized for 1 h at room temperature with 200 µL solubilization buffer (8 M Urea, 2% (wt/vol) CHAPS, 5 mM TBP, 20 mM TrisHCl pH 8.0), then alkylated by adding 7 µL of 0.5 M iodoacetamide and incubated for 1 h at room temperature in the dark. Then, 1150 µL of ice-cold methanol was added and samples were incubated for 2 hours at −20 °C. After centrifugation at 16,000 g at 4 °C, supernatant was discarded, and the pellet was re-solubilized in 160 µL rehydration buffer (7 M urea, 2 M thiourea, 1% (wt/vol) DTT, 1% (wt/vol) CHAPS, 1% (wt/vol) Triton X-100, 1% (vol/vol) ampholyte, trace amount of Bromophenol Blue). Fixed immobilized pre-cast IPG strips (cat. #ZM0018, Life Technologies, Carlsbad, CA) with a linear pH gradient 3–10 were rehydrated in 155 µL of the resulting mixture overnight at room temperature inside IPG Runner cassettes (cat. # ZM0008, Life Technologies). Isoelectrofocusing (first dimension separation) was performed at room temperature with rising voltage (175 V for 15 minutes, then 175–2,000 V linear gradient for 45 minutes, then 2,000 V for 30 minutes) on Life Technologies Zoom Dual Power Supply using an XCell SureLock Mini-Cell Electrophoresis System (cat. #EI0001, Life Technologies). The IPG strips were then equilibrated for 15 minutes consecutively in (i) 6 M Urea, 20% (vol/vol) glycerol, 2% SDS, 375 mM Tris-HCl pH 8.8, 130 mM DTT and (ii) 6 M Urea, 20% (vol/vol) glycerol, 2% SDS, 375 mM Tris-HCl pH 8.8, 135 mM iodoacetamide, and loaded on 4–12% Bis-Tris ZOOM SDS-PAGE pre-cast gels (cat. # NP0330BOX, Life Technologies). For the second dimension, SDS-PAGE was performed for 1 h at 170 V. Immunoblotting was performed as described above.

### Statistical analysis

Results are presented as the mean ± Standard Deviation. Statistical significance (P) between groups were calculated by Student’s t-test using Microsoft excel, and indicated as * for value of statistical significance (P < 0.05), ** for value of statistical significance (P < 0.005) and ^#^ for insignificant (P > 0.05) statistics.

## Electronic supplementary material

Supplemental data
