# Supplementary material for: Inflammatory response of microglia to prions is controlled by sialylation of PrPSc
Source: Sci Rep. 2018 Jul 27;8:11326. doi: 10.1038/s41598-018-29720-z (PMC6063910; doi:10.1038/s41598-018-29720-z)
Supplement: Supplementary file 1 — Supplemental data [file 41598_2018_29720_MOESM1_ESM.pdf]

Supplementary material

Inflammatory response of microglia to prions is controlled by sialylation of PrP^{Sc}

Saurabh Srivastava[1,2]*, Elizaveta Katorcha[1,2], Natallia Makarava[1,2], James P. Barrett[3], David J. Loane[3], Ilia V. Baskakov[1,2 #]

[1] Center for Biomedical Engineering and Technology, University of Maryland School of Medicine, Baltimore, Maryland, 21201 United States of America, [2] Department of Anatomy and Neurobiology, University of Maryland School of Medicine, Baltimore, Maryland, 21201 United States of America, [3] Department of Anesthesiology and Shock, Trauma and Anesthesiology Research (STAR) Center, University of Maryland School of Medicine, Baltimore, Maryland, 21201 United States of America

[#] To whom correspondence should be addressed: Center for Biomedical Engineering and Technology, University of Maryland School of Medicine, Baltimore, 111 S. Penn St., Baltimore, MD 21201. Phone: 410-706-4562; FAX: 410-706-8184. Email: Baskakov@umaryland.edu

*Current address: Department of Cellular and Molecular Medicine, University of California, San Diego, La Jolla, California, 92093, United States of America

Supplemental Figure Legends.

Figure S1. Purification and 2D analysis of PrP^{Sc}. (A) Analysis of purified, brain-derived 22L PrP^{Sc} by silver staining (left panel) and Western blot using anti-PrP Ab3531 antibody (right panel). Full-length recombinant PrP is shown as references of leading amounts. (B) Analysis of PrP^{Sc} by 2D Western blot: 22L scrapie brain homogenate (i); brain-derived, purified 22L PrP^{Sc} mock-treated using conditions for neuraminidase treatment (ii); brain-derived, purified 22L PrP^{Sc} treated with neuraminidase (iii); ScN2a cell-derived, purified 22L PrP^{Sc} mock-treated using conditions for neuraminidase treatment (iv); ScN2a cell - derived, purified 22L PrP^{Sc} treated with neuraminidase (v). (C) Assessing the amounts of PrP^{Sc} in purified preparations of 22L and ds22L in comparison to 1% 22L scrapie brain homogenate. All Western blots were stained using Ab3531 antibody.

Figure S2. Analysis of inflammatory responses of BV2 cells to neuraminidase treatment. To examine whether the neuraminidase or the buffer components used for the desialylation reaction have any inflammatory effects, BV2 cells were treated with neuraminidase (Neu), heat denatured neuraminidase (D.Neu) or Glycobuffer1 (the buffer used for neuraminidase treatment reactions) using experimental conditions identical to the experiments presented in Figures 2 and 3. The final concentration of neuraminidase in cultured cells was the same as in the experiments on treatment of BV2 cells with ds-22L PrP^{Sc} material presented in Figure 2 and 3. Levels of TNFα (A), IL6 (B), NO (C) and iNOS were measured upon treatment of BV2 cells for 18 hours. In parallel, cells were cultured in the absence of treatment (ctrl) or were treated with LPS (100 ng/ml). In panel C, the concentration of NO in fresh culture media is marked a as Blank. Expression levels of iNOS were normalized relative to the intensity of the β actin.

Figure S3. Dose dependent effect of 22L and ds-22L on NO production in BV2 cells. A dose response of BV2 cells exposed to purified 22L or ds-22L PrP^{Sc} for 18 hours, as analyzed by measuring NO secretion. NO secretion in non-treated control is shown for reference (ctrl). The final concentrations of 22L or ds-22L PrP^{Sc} in the cell culture media spike with 10 μl of purified material were equivalent to the amounts of PrP^{Sc} in 0.75% 22L scrapie brain homogenate.

Figure S4. Full-length Western blots shown in Figure 3A and Figure 6A.

Figure S5. Full-length Western blots shown in Figure 4A.

Figure S1

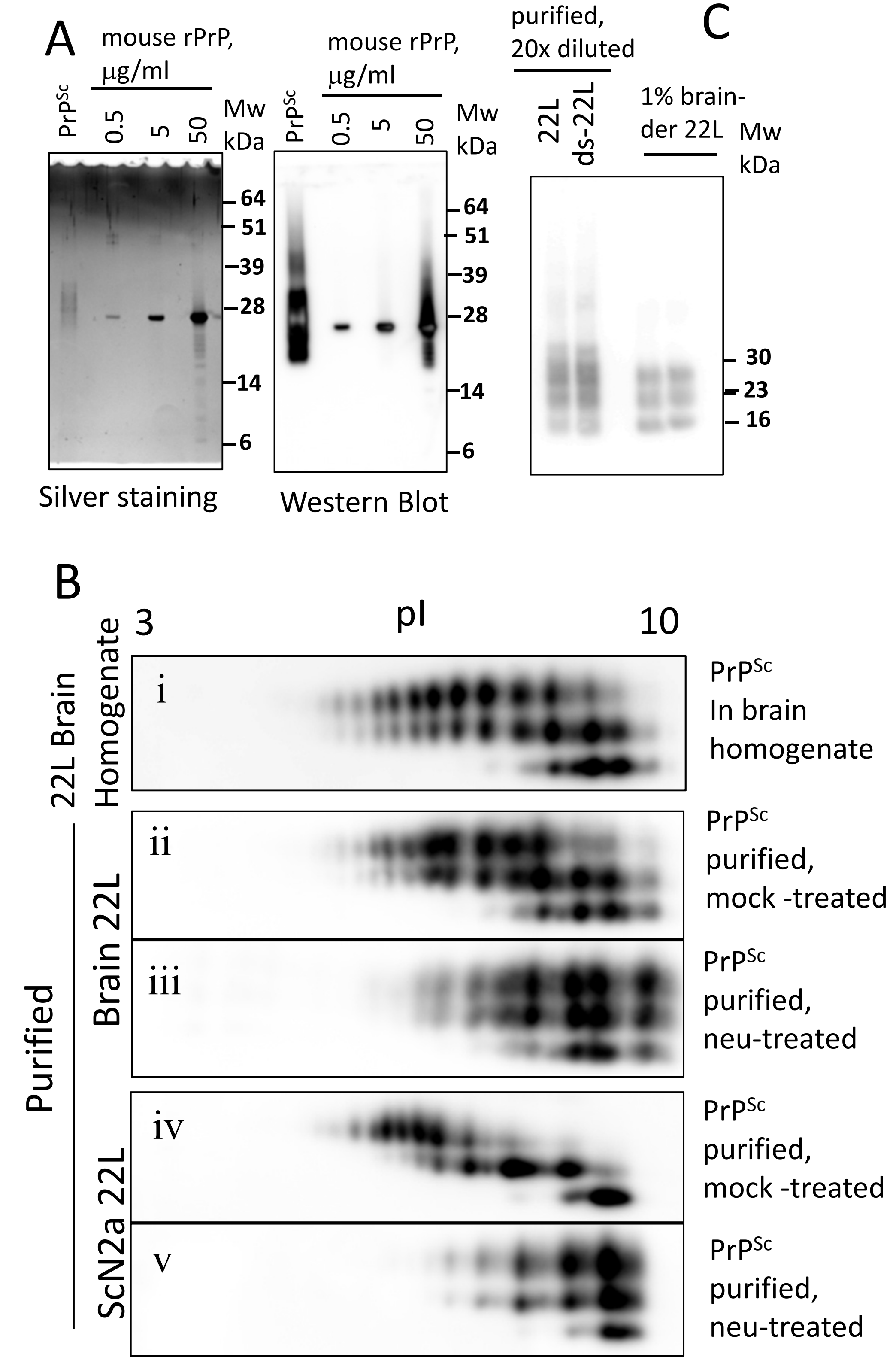
A
mouse rPrP, μg/ml
PrPSc
0.5
5
50
Mw kDa
64
51
39
28
14
6
Silver staining
Western Blot
C
purified, 20x diluted
22L
ds-22L
1% brain-der 22L
30
23
16
B
3
pI
10
22L Brain Homogenate
i
PrPSc In brain homogenate
Purified
Brain 22L
ii
PrPSc purified, mock -treated
iii
PrPSc purified, neu-treated
ScN2a 22L
iv
PrPSc purified, mock -treated
v
PrPSc purified, neu-treated

Figure S2

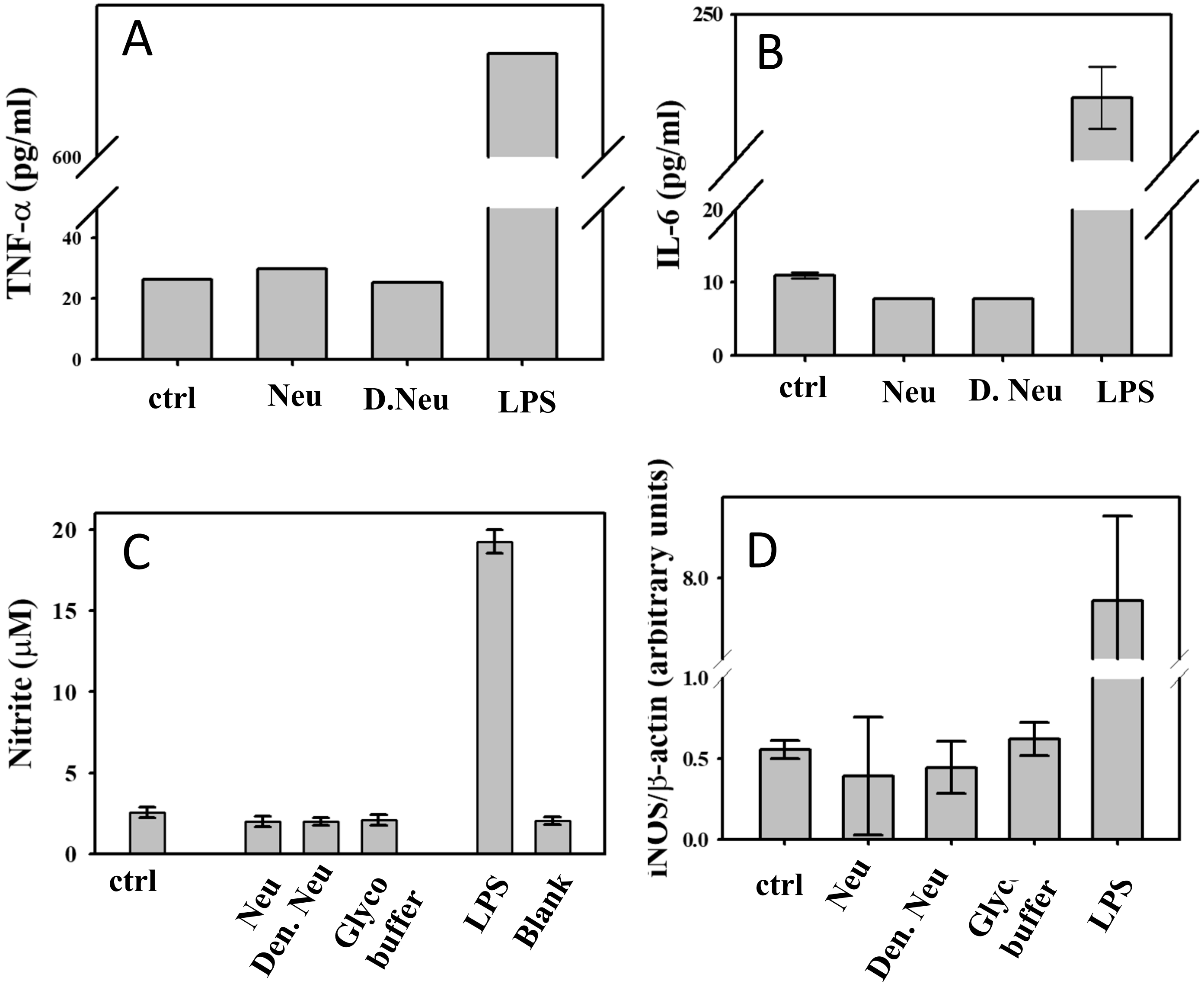
A
TNF-α (pg/ml)
600
40
20
0
ctrl
Neu
D.Neu
LPS
B
IL-6 (pg/ml)
250
20
10
0
ctrl
Neu
D. Neu
LPS
C
Nitrite (μM)
20
15
10
5
0
ctrl
Neu
Den. Neu
Glyco buffer
LPS
Blank
D
iNOS/β-actin (arbitrary units)
8.0
1.0
0.5
0.0
ctrl
Neu
Den. Neu
Glyco buffer
LPS

Figure S3

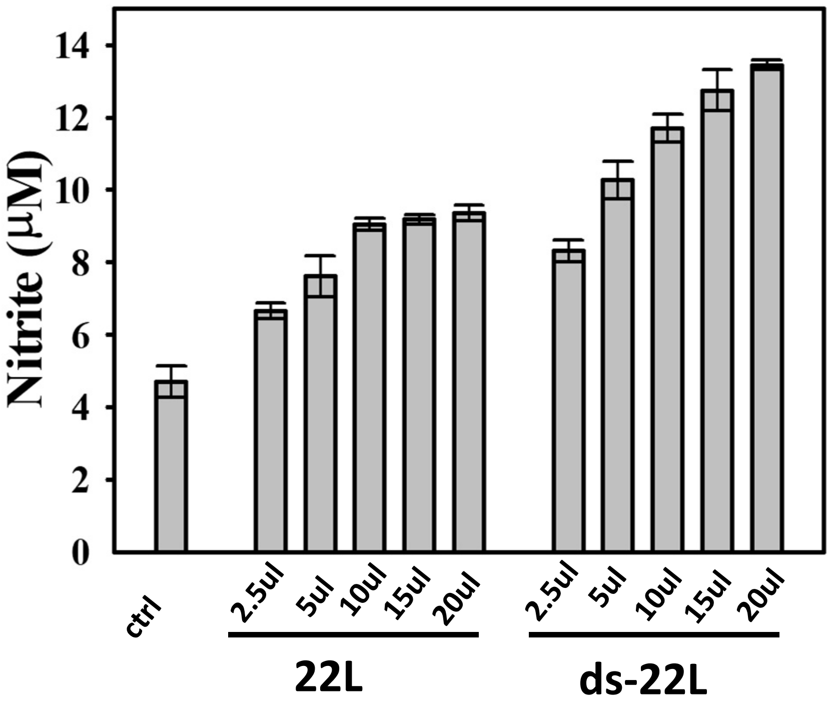

Nitrite (μM)
14
12
10
8
6
4
2
0
ctrl
2.5ul
5ul
10ul
15ul
20ul
2.5ul
5ul
10ul
15ul
20ul
22L
ds-22L

Figure S4

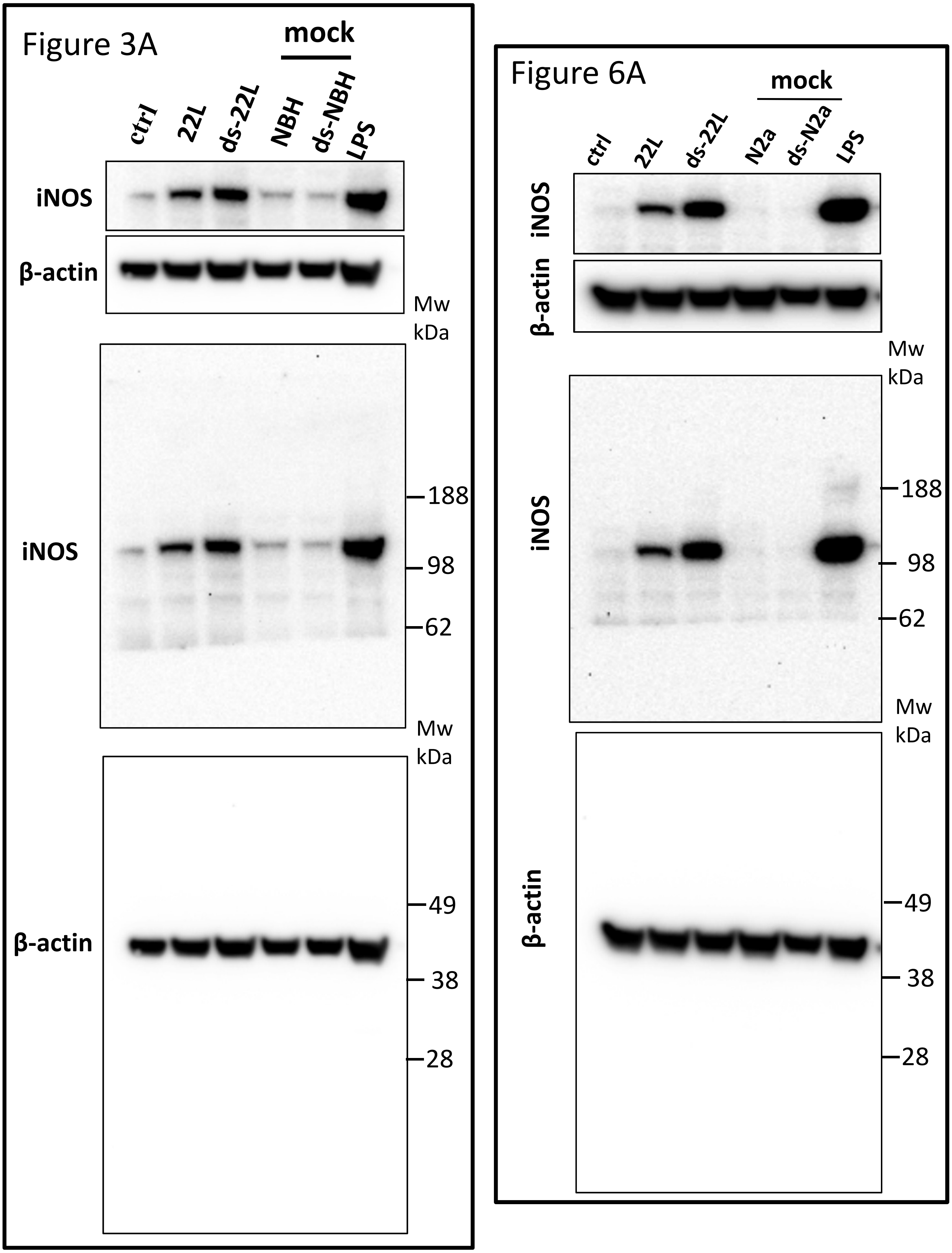
Figure 3A
mock
ctrl
22L
ds-22L
NBH
ds-NBH
LPS
iNOS
β-actin
Mw kDa
188
98
62
49
38
28
Figure 6A
mock
ctrl
22L
ds-22L
N2a
ds-N2a
LPS
iNOS
β-actin
Mw kDa
188
98
62
49
38
28

Figure S5

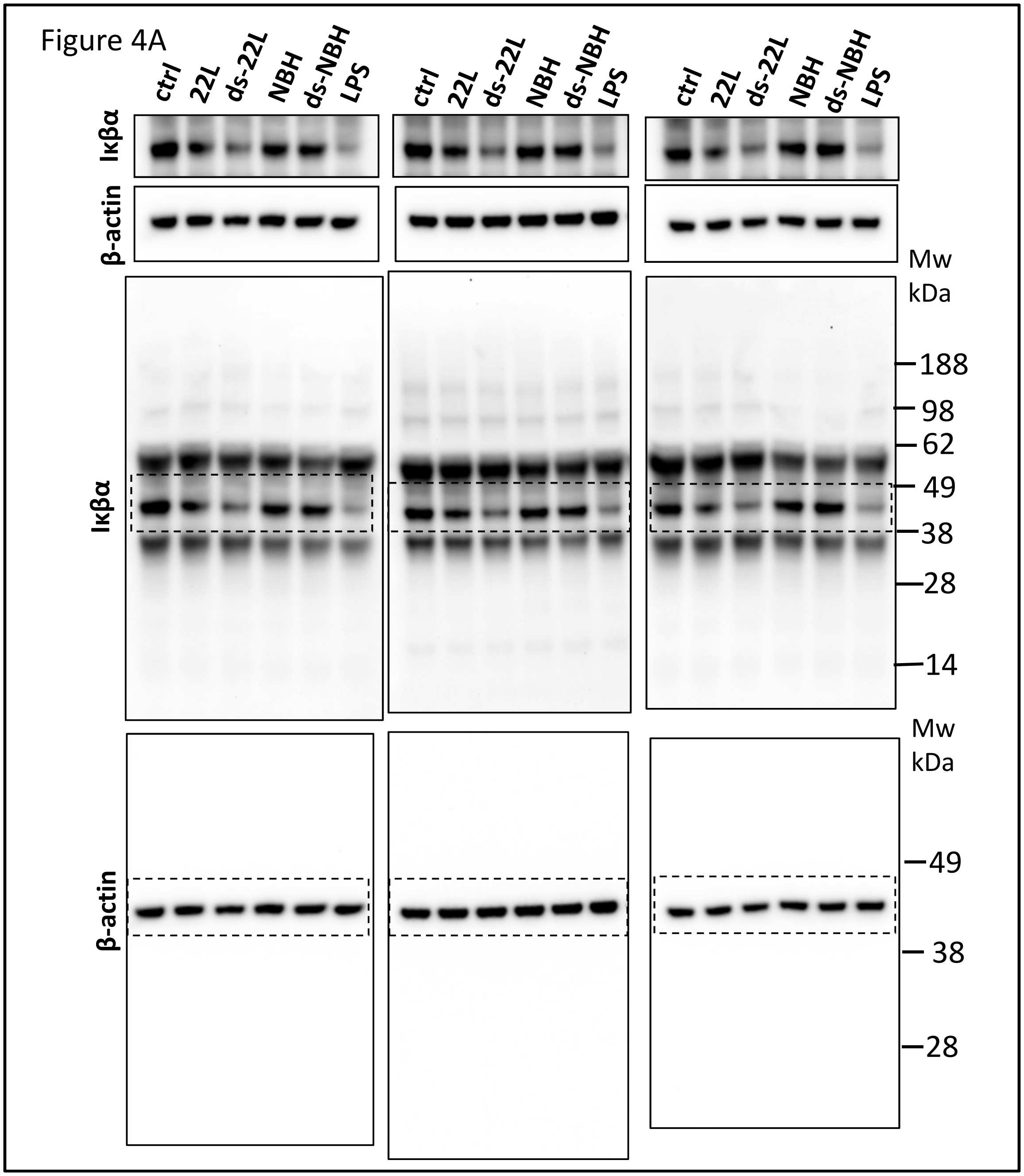

Figure 4A
ctrl
22L
ds-22L
NBH
ds-NBH
LPS
Iκβα
β-actin
Mw
kDa
188
98
62
49
38
28
14
Iκβα
Mw
kDa
49
38
28
β-actin
